# Increased prevalence of rotavirus gastroenteritis among children in Riyadh Saudi Arabia

**DOI:** 10.1186/1753-6561-5-S6-P48

**Published:** 2011-06-29

**Authors:** HT Tayeb, HH Balkhy, S Aljuhani, EA Albanyan, S Alalola, M AlShaalan

**Affiliations:** 1 King Abdulla International Medical Research Center, KAMC, Riyadh, Saudi Arabia; 2 Infection Prevention and Control Department, KAMC, Riyadh, Saudi Arabia; 3 Division of Microbiology, Department of Pathology and Laboratory Medicine, KAMC, Riyadh, Saudi Arabia; 4 Department of Pediatrics, KAMC, Riyadh, Saudi Arabia

## Introduction / objectives

This research aims to study the epidemiology of human rotavirus (HRV) in pediatric patients at a tertiary care hospital in Riyadh, KSA.

## Methods

One thousand and seven diarrheal stool samples had been prospectively collected Between Jan1^st^, 2008 and OCT 31^st^, 2010 period from hospitalized patients below the age of 5 year with none bloody, none chronic diarrhea. Samples were examined using ELISA for rotavirus. Demographic data were collected.

## Results

HRV was detected in 65.5% (660/1007). There was a significant difference between males and females acquiring the disease (57.5%, 380/660 vs 42.4%, 280/660, respectively. *P* value <0.05). Children who were 1 year of age or less had more infection than those who were over 1 year of age (81%, 534/660 vs. 19%, 126/660, respectively; *P*=0.0005). Infections occured throughout the year with no clear significant seasonal peaks, figure 1.

## Conclusion

The high rate of positivity, are at variance with previously published reports of rotavirus infection in Saudi Arabia, since 2005. This may be explained by improvements in public health introduced over the past 20 years. Our increasing rate however, of 65.5% may suggest the emergence of new serotypes, not present in our populations in earlier reports. Further molecular testing is needed to prove such hypothesis.

## Disclosure of interest

None declared.

